# Transcriptome sequencing reveals circRNA expression profile in Parkinson’s disease-like mice after aerobic exercise

**DOI:** 10.55730/1300-0152.2611

**Published:** 2022-03-21

**Authors:** Tianlun FAN, Xiating LI, Chuan FU, Lichun SUN

**Affiliations:** The First Affiliated Hospital of Hainan Medical College, Hainan, China

**Keywords:** Parkinson’s disease, aerobic exercise, behavioral deficits, circZfp827, circHivep2, circTshz2

## Abstract

Parkinson’s disease (PD) is a common complex neurodegenerative disease, and aerobic exercise (EX) has potential to improve motor dysfunction. This study aimed to explore whether EX acts on PD in mice mode. Mice were administered 1-methyl-4-phenyl-1,2,3,6-tetrahydropyridine (MPTP) and subjected to a 4-week physical exercise regimen (EX-PD group) and underwent RNA-Seq. Here, MPTP caused PD, which was characterized by neuron shrinkage and behavioral deficits, whereas EX improved PD by rescuing neuronal survival and motor function in mice. Moreover, circRNA expression profiles identified a total of 142 differentially expressed circRNAs (DEcircRNAs) between PD and EX-PD group. These DEcircRNAs were mainly involved in PD, dopaminergic synapses, and calcium signaling pathways. The expression of circZfp827 and circTshz2 were significantly elevated in PD group while reduced owing to EX intervention. In contrast, EX intervention significantly restored decline in circHivep2 expression due to PD. The circRNA-miRNA-mRNA network suggested that circZfp827, circHivep2, and circTshz2 were involved in ceRNA mechanism of EX to improve PD, and their target genes were significantly decreased after interference. The directly binding regulation relationship of circTshz2-mmu-miR-326-3p-Th was verified by double luciferase reporter assay. Our research revealed that EX improved motor behavioral deficits and pathological features of PD mice, and circRNA-based signatures are potential candidates for further assessment as PD biomarkers for improvement by EX.

## 1. Introduction

Parkinson’s disease (PD) plagues approximately 2% of the world’s elderly and is the second most common neurode-generative disease after Alzheimer’s disease (AD) (Tysnes and Storstein 2017). The pathological features of PD are mainly insoluble Lewy bodies formed by the accumulation of α-synuclein (SNCA) and the selective loss of dopaminergic neurons in the nigrostriatal (Marques and Outeiro 2012). Another central pathological feature of PD is neuroinflammation within the central nervous system (CNS), which is involved in astrocytes, neurons, and microglia. Abnormalities in the blood-brain barrier (BBB) facilitate neuroinflammation, leading to synaptic disruption and neuronal death, aggravating the progression of PD (Desai et al., 2007). In short, whether it is Lewy bodies, neuroinflammation, or abnormalities of the BBB, they are closely related to PD, constituting the complex and multifactorial pathogenesis of PD and introducing obstacles to its treatment. Fortunately, in recent years, studies have found that exercise is not only beneficial for the whole body but also improves cognitive function and brain health to alleviate PD (Gomes-Osman et al., 2017).

Exercise is a systemic behavior. Both aerobic exercise (EX) and strength exercise can interfere with dopaminergic signaling and improve cognitive and motor dysfunction, including tremor, delayed behavior, and decreased memory and depression (Ridgel et al., 2012; Fisher et al., 2013; Wu et al., 2017). Real et al. (2017) found that 4 consecutive weeks of treadmill exercise significantly reduced the neuroinflammatory response and diminished activation of astrocytes, microglia, and oxidants, suggesting that exercise can significantly decrease the incidence of PD (Real et al., 2017). Moderate intensity intermittent physical activity increases the level of brain-derived neurotrophic factor secretion in patients and alleviated neuroinflammation, thereby improving PD (Zoladz et al., 2014). However, the mechanism by which exercise improves PD remains unclear. Liu et al. (2019) believed that regular EX regulated the target calcium ion (Ca2+)/calmodulin-dependent protein kinase signaling pathway by activating miR-3557/324 to delay neurodegenerative disease and lesions (Liu et al., 2019). Koo et al. (2017) showed that treadmill exercise improved PD by regulating the Toll-like receptor 2/myeloid differentiation factor-88 adaptor protein/nuclear factor-**κB** signaling pathway (Koo et al., 2017). Although scholars have frequently explored the mechanism underlying PD improvement by exercise, they still have not resolved the issues surrounding PD treatment. Therefore, it is extremely urgent to discover new and key molecules during the progression of PD.

Circular RNAs (circRNAs) are a class of noncoding RNAs with a circular structure. Nowadays, scholars are indeed fully aware that mammalian genomes produce a portion of nontranscribed circRNAs, which have specific structures and/or regulatory functions (D’Ambra et al., 2019). In particular, circRNAs are mainly expressed in the brain and accumulate with age, especially at the site of neuronal activity and the synapses (Piwecka et al., 2017). Many pieces of evidence indicate that circRNAs play an important role in age-related neurodegenerative diseases, including AD and PD (Gokool et al., 2020). Wang et al. (2018) identified that circRNA participated in the process of Aβ1-42-induced AD in the form of competitive endogenous RNA (ceRNA) within the hippocampus (Wang et al., 2018). Study established that circDLGAP4 plays a neuroprotective role by modulating the miR-134-5p/cyclic response element-binding protein pathway to inhibit PD both in mice and humans (Feng et al., 2020). Then, ciRS-7 (also known as cdrlas) may modulate SNCA via a miR-7-dependent pathway, and circSNCA can directly sponge miR-7 to upregulate the expression of SNCA mRNA in a PD model (Hansen et al., 2013; Sang et al., 2018). Moreover, circBBS9 may play an active role in aging-associated muscle dysfunction by mediating EX (Guo et al., 2020a). Nevertheless, the function and mechanism of circRNAs generated during EX to improve neurodegenerative diseases, such as PD, requires additional evaluation.

In recent years, EX has been regarded nearly in consensus by researchers to substantially improve the progression of PD. Interestingly, circRNA is not only reported to be widely involved in PD progression, but is also an important small molecule that the organism actively responds to EX (Guo et al., 2020b). Therefore, this study aimed to explore that whether EX for improving PD is mediated by circRNA. 1-methyl-4-phenyl-1, 2, 3, 6-tetrahydropyridine (MPTP) is a drug that causes the loss of nigrostriatal dopaminergic neurons and is widely used to study the pathophysiological changes in PD (Meredith and Rademacher, 2011). We investigated the changes in neurons and motor injuries in PD mice (stimulated by systematic administration of MPTP before and after EX intervention), and explored the expression differences of circRNAs between the PD and aerobically exercised mice.

## 2. Materials and methods

### 2.1. Ethics statement and mice

All mice experiments were approved by the Institutional Ethics Committee of the First Affiliated Hospital of Hainan Medical College and we tried our best to alleviate the pain of all mice. All procedures were per the Guidance on the Care of Laboratory Animals. Male C57BL/6J mice aged 10–12 weeks (n = 18) were purchased from Shanghai Yingbio Tech (Shanghai, China). All mice were housed in natural light at room temperature for 1 week and were given access to water and food ad libitum.

### 2.2. Experimental design

All mice were randomly divided into three equal groups: sham-operated group (Sham, n = 6), PD group (PD, n = 6), and PD + EX group (EX-PD, n = 6). Mice in the sham group received a continuous intraperitoneal injection of 0.9% sodium chloride solution for 7 days. Mice in the PD and EX-PD groups underwent the same process using 0.9% saline injections, except that 0.33% MPTP (M9049, AbMole, Shanghai, China) was contained in 0.9% saline. After modeling with MPTP, all mice underwent behavioral testing and pathological testing to confirm the PD model.

### 2.3. Aerobic exercise interventions in mice

The MPTP-injected mice were placed in the same environment as before the operation and allowed to recover for 4 weeks. During rehabilitation, the sham and PD group mice received no specific training, while the EX-PD group mice received EX. The EX method included self-built drum-mesh training and self-made balance beam training. The method of self-built drum mesh training (Si et al., 2016) was as follows: the self-built drum-mesh trainer (self-built cylindrical net device with a length of 1.0 m and a diameter of 0.6 m) was divided into four cages with four mice that were trained at the same time for training mice in griping, rotating, walking, balancing, and other functions. The base has a fixed frame with a handle at one end, and the revolving speed was maintained at 5 revolutions per minute by pressing the handle. This self-built drum mesh training was conducted once a day, 30 min/day and 6 days/week, for 4 weeks.

The method of self-made balance beam training (Cai et al., 2017a) was as follows: a 170-cm long and 2-cm wide square wooden rod was laid flat at 7 cm away from the ground as a balance beam. Mice were allowed to walk for training the balance coordination function. Balance beam training was always followed by drum mesh training.

### 2.4. The forced swim test (FST) and pole test of motor behavior

The FST was used to assess antidepressant-like behavior in mice. The FST was performed at 0, 2, and 4 weeks after successful modeling. The swim cylinders (20 × 20 × 50) to 30 filled with water (23–25 °C) to obtain a record of the mice swimming in the cylinders for 1 min. The score was as follows: lasted swimming scored 3 points; occasionally floating scored 2 points; floating on the side of the body and only occasionally swam on the hind legs scored 1 point; and sinking of the hind limbs scored 0 points. Finally, the total score of each mouse was calculated.

The pole test was used to evaluate the degree of bradykinesia in mice. The pole test was performed at 0, 2, and 4 weeks after successful modeling. Mice were placed on top of a ball (with a diameter of 2.5 cm) of a pole 55 cm high and 1 cm in diameter. The wooden pole and ball were wrapped with gauze to prevent slipping. The mice were placed upside down on the ball, and the time required for the mice to complete the following movements was recorded: a. The time it took for the mice to climb down from the ball and finish the full length of the wooden pole; b. The time it took for the mice to climb the upper half of the wooden pole; and c. The time it took for the mice to climb the lower half of the wooden pole. Finally, the average was obtained by adding the time served as the final result of the test. If the mice stopped or crawled in the reverse direction, they were not recorded and were remeasured.

### 2.5. Sample collection

All mice were sacrificed at the end of the motor behaviors test for collected specimens of the brain tissues. Mice were euthanized via CO_2_ inhalation followed by decapitation and the brain tissues were removed. Tissue samples were stored at −80°C until they were ready for use in circRNA sequencing, hematoxylin–eosin (HE) staining, and quantitative reverse transcription PCR (RT-qPCR).

### 2.6. HE staining

The striatum tissues (n = 3) of the 3 groups of mice were immersed in 4% formaldehyde solution and fixed for 48 hours. The samples were then dehydrated conventionally and embedded in paraffin. Subsequently, the striatum tissues were cut into 3-mm thick continuous sections for HE staining. Pathological changes in nerve cells were observed under an optical microscope (BX-43; Olympus, Tokyo, Japan).

### 2.7. Total RNA isolation

Total RNA was extracted with TRIzol reagent (e) from PD (n = 3) and EX-PD (n = 3) mouse striatum tissues according to the manufacturer’s protocol. The concentration and purity of RNA were measured using a microspectrophotometer (Tiangen Biotech Co., Ltd., Beijing, China). Quality qualified RNA was frozen at −80°C for subsequent experiments.

### 2.8. CircRNA-Seq and bioinformatics analyses

The total RNA prepared as described earlier was used for circRNA sequencing. Adapters were added to both ends of the primers and amplified the cDNA library (Collibri Library Amplification Master Mix, Thermo, USA). Subsequently, the constructed cDNA library was qualified using an Agilent 2100 Bioanalyzer and ABI Step One Plus real-time PCR System, and sequenced on an Illumina HiSeq 2500 platform (Illumina, San Diego, USA) with a 150-bp paired-end run. The raw reads were qualified using FastQC (http://www.bioinformatics.babraham.ac.uk/projects/fastqc/) and retrieved filtering data were mapped to the human reference genome (GRCm38). The ACFS2 (https://github.com/arthuryxt/acfs) was used to identify circRNAs, and the fragments per kilobase per million (FPKM) were used to normalize expression of circRNAs. CircRNAs that the expression between in the PD group and EX-PD group to meet the condition of log2FC > 1 or < −1 and false discovery rate (FDR) < 0.05, which was defined as a differentially expressed circRNA (DEcircRNA) using the DESeq2.0 algorithm. All the DEcircRNAs were used to predict the target genes and then mapped to the Gene Ontology (GO) database and Kyoto Encyclopedia of Genes and Genomes (KEGG) database to obtain functional annotation.

### 2.9. Verification of RT-qPCR

The RNA was isolated as described before, and reverse transcribed into the First-strand cDNA Using RevertAid First Strand cDNA Synthesis kit (ThermoScientific, Madison, USA), and then amplified to cDNA using FastStart Universal SYBR Green Master mix with QuantStudio 6 Flex Real-Time PCR System (Thermo Fisher Scientific, Inc.) according to the manufacturer’s instructions. The PCR program was as follows: 95 °C for 10 min; 95 °C for 15 s, 60 °C for 60 s, and repeated for 45 cycles. All primers used in this study were synthesized by Sangon Biotech (Shanghai, China) and shown in Table S1. GAPDH, β-Actin and B2m were served as an internal reference gene and the relative gene expression was determined by the 2^−ΔΔCq^ method (Livak and Schmittgen 2001).

### 2.10. The circRNA-miRNA-mRNA ceRNA network

The MiRanda and TargetScan algorithms were used to predict the target miRNAs of the five DEcircRNAs, and the intersection of the two algorithms was taken as the final result. The target genes for miRNAs were then predicted using the miRWalk 3.0 database. All miRNAs, mRNAs, and 5 DEcircRNAs were subjected to Cytoscape software to construct a network.

### 2.11. Cell culture and transfection

The mouse hippocampal neuron cell line of HT22 (CL-0595, Procell Life Science & Technology, China) was cultured in DMEM medium supplemented with 10% fetal bovine serum and 1% penicillin-streptomycin solution and then placed in incubator at 37 °C with 5% CO_2_. The sequence of interfering RNAs (siRNAs) were synthesized by Gibico (Shanghai, China), shown in Table S1. All these siRNAs were transfected into HT22 cells using Lipofectamine 2000 (Invitrogen), according to the manufacturer’s instructions. Finally, the transfection efficiency was measured by RT-qPCR.

### 2.12. Double luciferase reporter assay

To verify the regulation relationship between circRNA and mRNA on the basis of ceRNA mechanism, we performed double luciferase reporter assay. The mRNA or circRNA fragments containing wild-type or mutant (MUT) binding sites on mmu-miR-326-3p were synthesized (GenePharma, Shanghai, China) and cloned into the psiCHECK-2 luciferase vector (Promega). These recombinant plasmids were sequenced and cotransfected with the sequences of mmu-miR-326-3p mimics or the mimics NC into 293T cells using Lipofectamine^TM^ 2000 according to the manufacturer’s instructions. The sequence of mmu-miR-326-3p mimics and NC were synthesized by Gibico (Shanghai, China). After 5 h of culture, the fresh medium was replaced and then cultured for another 48 h. Finally, the fluorescence was detected by dual-luciferase kit (Promega), and the relative value of luciferase activity was conducted from firefly luciferase activity value/renilla luciferase activity value.

### 2.13. Statistical analysis

The Statistical Package for the Social Sciences (SPSS; Chicago, USA) version 17.0 software was employed for statistical analysis and the data were presented as the mean ± SD. Statistically significant differences were analyzed by one-way analysis of variance (ANOVA) followed by Tukey’s test. P < 0.05 was considered statistically significant.

## 3. Results

### 3.1. Aerobic exercise improves behavioral deficits and pathological features of PD

**Figure 1:**
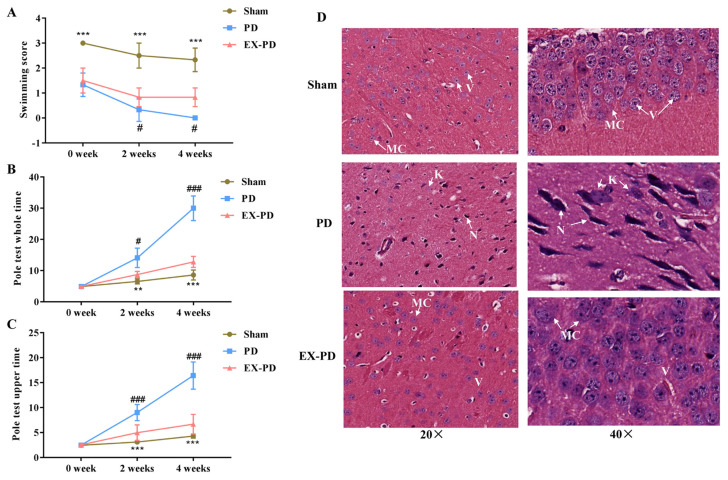
Aerobic exercise improves behavioral deficits and pathological features of PD. A. The average score of mice (n = 6) with the FST test. B. The average time for mice (n = 6) in terms of finished climbing the whole of the wooden pole. C. The average time of mice (n = 6) for finished climbing the upper half of the wooden pole. D. HE staining of neurons in the hippocampus. The neuronal cells from sham group were neatly arranged and showed scattered large multipolar neurons (MC) with vesicular nuclei (V); MPTP induced neuronal degeneration, in the PD group, showed dark stained cytoplasm (N) or karyolytic nucleus (K). EX significantly improved damage change induced by MPTP, in EX-PD group, neuronal structure similar to that of the sham group. All data were analyzed by one-way ANOVA followed by Tukey’s test. * represents the PD group compared with sham group, and p < 0.05 was indicated by *, p < 0.01 was indicated by **, and p < 0.001 was indicated by ***. # represents that the aerobic exercise PD (EX-PD) group compared with the PD group, and p < 0.05 was indicated by #; p < 0.01 was indicated by ##; p < 0.001 was indicated by ###.

The results of FST showed that after 4 weeks of modeling, the score in the sham group did not significantly decrease, but the score in the PD group fell to 0, suggesting that depression-like behavior occurred in mice. In addition, EX intervention had a significant antidepressant effect (Figure 1A). With the pole test, in the course of climbing the whole wooden pole, the motor function of PD mice was gradually lost which was characterized by prolonged time, while EX-PD mice experienced a rescue of part of their motor ability (Figure 1B). Similarly, climbing the upper half of the wooden pole, with the prolongation of the modeling time, EX significantly alleviated the dyskinesia of PD mice (Figure 1C). Neuronal damage was assessed by HE staining, and we observed the protective effects of EX on neuronal injury in the mouse brain tissues after PD. As shown in Figure 1D, the neuronal cells from the sham group were neatly arranged and showed scattered large multipolar cells with vesicular nuclei. However, the PD group showed degeneration of most neuronal cells that had deeply stained cytoplasm or karyolytic nucleus. In contrast to the PD group, neuronal structures were significantly improved in the EX-PD group, showing scattered large multipolar cells with vesicular nuclei and regularly stained nuclei similar to that of the sham group (Figure 1D). These results indicate that EX improves behavioral deficits and the pathological features of PD in mice.

### 3.2. Illumina sequencing output and quality control

To analyze the changes in circRNA expression profile of PD mice after EX intervention, three samples from the PD group and the EX-PD group were used for RNA sequencing. As shown in Table S2, a total of 55–116 million clean reads were output with a filtering ratio between 0.947 and 0.960. The GC content and mapped rate of reads were between 44%–46% and 0.915–0.946, respectively, which indicated that the quality of sequencing data was trustworthy.

### 3.3. Analysis of DEcircRNA

**Figure 2:**
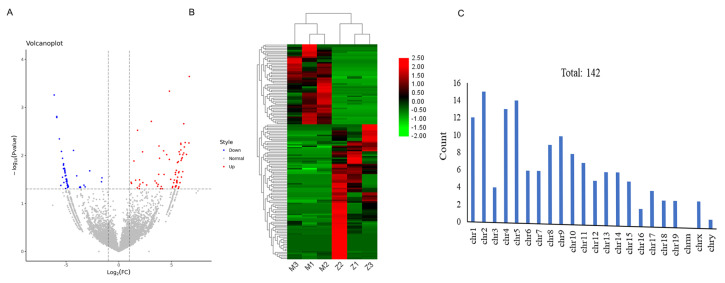
Analysis of the DEcircRNAs between the PD group and aerobic exercise PD (EX-PD) group. A. Volcano plot of DEcircRNAs. Red dot represents DEcircRNAs upregulated and the blue dot represents DEcircRNAs downregulated in the EX-PD group compared with the PD group. B. Cluster heatmaps of significant DEcircRNAs. Z represents the sample that was from the EX-PD group and M represents the sample from the PD group. Each column represents a sample, and the row shaded in red represents upregulated while that shaded in green represents downregulated when EX-PD *vs* PD. C. The distribution of DEcircRNAs on chromosomes (chr.).

Screening differentially expressed genes is helpful for mining key genes in different biological backgrounds. In this study, 142 DEcircRNAs were identified between the PD group and the EX-PD group. Among them, there were 89 upregulated and 53 downregulated circRNAs in the EX-PD group compared with the PD group (Figure 2A). The distinguishable circRNA expression patterns between the six samples were shown by hierarchical cluster, and the figure showed that DEcircRNAs of the PD group and the EX-PD group were significantly clustered into two branches, as expected (Figure 2B). The genome distribution analysis indicated that these DEcircRNAs were mainly distributed in chr2, chr5, and chr4 (Figure 2C).

### 3.4. GO and KEGG pathway analysis of DEcircRNAs

**Figure 3:**
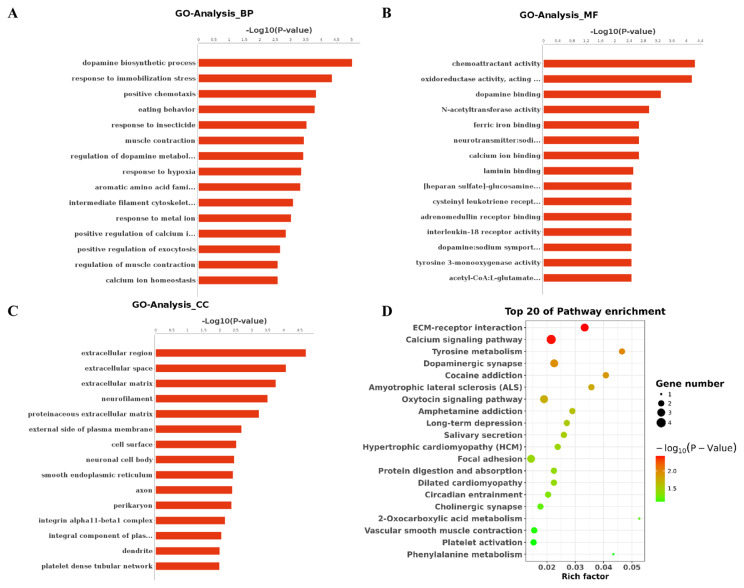
GO and KEGG pathway enrichment based on DEcircRNAs. A**:** The top 15 GO terms in three domains. A shows DEcircRNAs enriched into BP; B shows DEcircRNAs enriched into MF; C shows DEcircRNAs enriched into CC. The left represents the GO term while column represents the p-value. D: The top 20 KEGG enrichment items. The left represents the KEGG pathway, right represents enrichment, and the size of the solid circle indicates the number of genes.

To mine the key DEcircRNAs involved in EX regulating PD, all DEcircRNAs had undergone the analysis of GO and KEGG. The top 15 significantly different GO terms of three domains, cellular component (CC), molecular function (MF), and biological process (BP), were observed (Figure 3A–3C). Most of these GO terms were related to neuronal regulation or metabolism, such as dopamine biosynthetic process and regulation of dopamine metabolic process in BP, dopamine binding, neurotransmitter: sodium symporter activity and calcium ion binding in MF, and neuronal cell body neurofilament in CC. These results suggest that DEcircRNAs may be related to neuronal regulation or metabolism. Moreover, KEGG pathway analysis showed that the DEcircRNAs were mainly enriched in extracellular matrix (ECM)-receptor interaction, PD, dopaminergic synapse, calcium signaling pathway, and oxytocin signaling pathway (Figure 3D). These pathways were related to the regulation of PD, which implied that the EX alleviated PD progression may be involved in the aforementioned pathways by DEcircRNAs.

### 3.5. Validation of the DEcircRNAs

**Figure 4:**
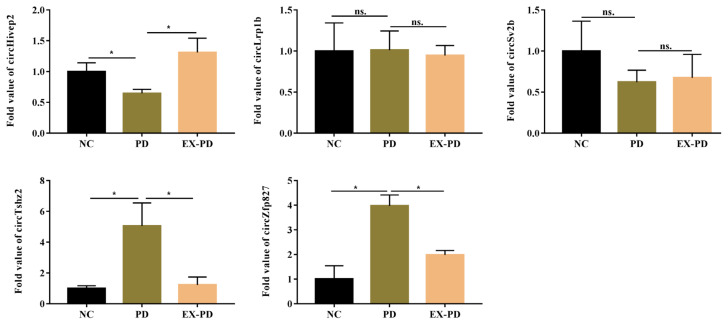
Validation of five selected DEcircRNAs. Gene expression of circHivep2, circLrp1b, circSv2b, circTahz2, and circZfp827 was normalized to GAPDH, β-Actin and B2m transcript levels. All experiments were repeated three times. One-way ANOVA was applied to analyze the data followed by Tukey’s test. * indicates the significant difference of p < 0.05, ns. indicates the significant difference of p > 0.05.

The five most significant circRNAs with highly expressed abundance were selected for RT-qPCR verification. In the RNA-seq data, compared with PD group, chr10_14066684_14004201_+62483-Hivep2 (circHivep2), chr7_75149088_75136298_-12790-Sv2b (circSv2b) and chr2_41113094_41110754_-2340-Lrp1b (circLrpib) were upregulated in the PD group, and chr2_169886586_169883526_+3060-Tshz2 (circTshz2) and chr8_79076538_ 79060250_+16288-Zfp827 (circZfp827) were downregulated in the EX-PD group. With the RT-qPCR results, the expression pattern between the PD group and EX-PD group of circHivep2, circTshz2, and circZfp827 were consistent with the RNA-seq results (Figure 4). In addition, compared with the sham group, circTshz2 and circZfp827 were upregulated and circHivep2 was downregulated in the PD group. However, the expression of circSv2b and circLrpib did not exhibit any difference between the three groups (Figure 4). Therefore, only circTshz2, circZfp827, and circHivep2 expression patterns were consistent with RNA-seq and they were used for subsequent study.

### 3.6. Prediction of circRNA-miRNA-mRNA ceRNA network

**Figure 5:**
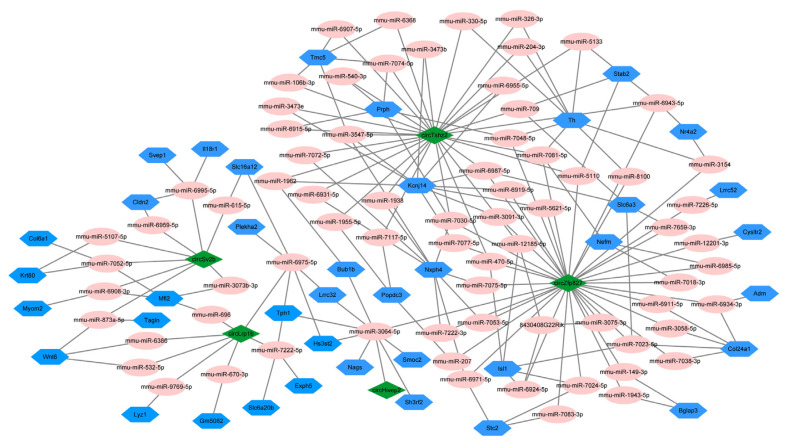
The ceRNA network of circRNA-miRNA-mRNA, including five circRNAs, 72 miRNAs, and 40 mRNAs. Green represents circRNA, pink represent miRNA, and blue represents mRNA.

In general, circRNA functions as a sponge for miRNA; hence, the potential target miRNAs were predicted and displayed by Cytoscape. As shown in Figure 5, the five DEcircRNAs predicted a total of 72 miRNAs and 40 mRNAs. All the predicted numerical scores (based on MiRanda and TargetScan algorithms) for these five DEcircRNAs were shown in Table S3. Interestingly, except for circHivep2 targeting to mmu-miR-3064-5p, the remaining four DEcircRNAs have at least seven targeted binding miRNAs, such as circLrpib-predicted targets of miR-532-5p, miR-6769-5p, miR-6386, miR-670-3p, miR-7222-5p, miR-696, and miR-6975-5p; the circTshz2 predicted 28 target miRNAs, including miR-330-5p, miR-204-3p, and miR-326-3p; circZfp827 predicted 35 target miRNAs, including miR-207 and miR-149-3p (Figure 5). Moreover, these 40 targeted mRNAs were related to the pathway of regulating PD progress or EX, such as collagen alpha-1 (XXIV) chain (Col24α1) and tyrosine 3-monooxygenase (Th), were predicted as potential target genes for circZfp827. Interestingly, Th also predicted that it might combine with miR-330-5p, miR-204-3p, and miR-326-3p (Figure 5). In brief, we speculated that these five DEcircRNAs may be related to EX improving PD via a ceRNA mechanism.

### 3.7. Verification of target gene expression

**Figure 6:**
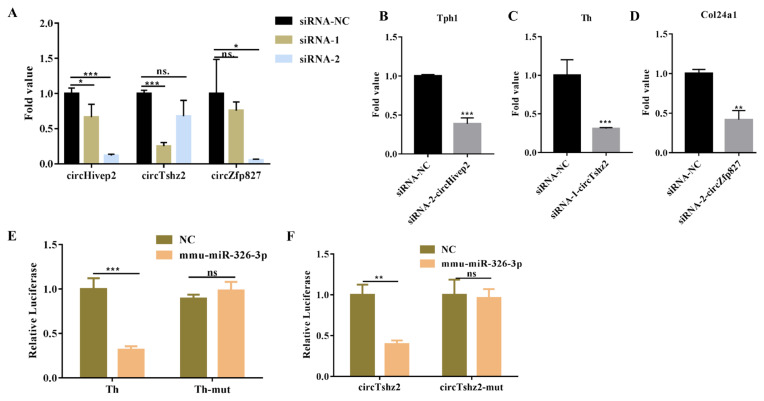
Verification of target gene expression after interfered circRNA expression. A. Interference efficiency detection of three circRNAs. B. The expression of predicted target gene Tph1 after interfered with circHivep2. C. The expression of predicted target gene Th after interfered with circTshz2. D. The expression of predicted target gene Col24α1 after interfered with circZfp827. E. The binding relationship between Th and mmu-miR-326-3p was verified by double luciferase reporter assay. F. The binding relationship between circTshz2 and mmu-miR-326-3p was verified by double luciferase reporter assay. Gene expression of Tph1, Th, and Col24α1 were normalized to GAPDH, β-Actin and B2m transcript levels. All experiments were repeated three times. t test, * p < 0.05, *** p < 0.001, ns. p > 0.05.

To further verify the regulatory relationship between the predicted target genes and the candidate circRNAs, we used siRNA to interfere with the expression of circRNAs, and then detected the expression level of target genes. Since only circTshz2, circZfp827, and circHivep2 showed significant differences between three groups in RT-qPCR results, and their expression patterns were consistent with RNA-seq, only the target gene of them was verified. As shown in Figure 6A, compared with siRNA-NC, siRNA-2-circHivep2, siRNA-1-circTshz2, and siRNA-2-circZfp827 could significantly down-regulate the expression of corresponding circRNA, so they were used in subsequent interference experiments. According to the results in Figure 5, circTshz2, circZfp827, and circHivep2 have multiple target genes, and we selected a gene that has high binding energy (MiRanda and TargetScan) and involved in neuronal regulation. For example, tryptophan 5-hydroxylase 1 (Tph1), Th, and Col24a1 were the potential target genes of circHivep2, circTshz2, and circZfp827, respectively (Figure 5), and they were reported to have impact on neuronal mechanisms (Haavik et al., 1997; Hubert et al., 2009; Zill et al., 2009). The study showed that interfered circHivep2 expression would cause a significant decrease in the expression of Tph1 (Figure 6B). Similarly, interference with the expression of circTshz2 and circZfp827 would also downregulate the expression of Th and Col24a1, respectively (Figures 6C and 6D). Thus, circHivep2, circTshz2, and circZfp827 respectively target downregulated the expression of Tph1, Th, and Col24a1 may be through the ceRNA mechanism. To further verify the above regulation relationship was ceRNA mechanism, double luciferase reporter assay was introduced to detect the direct binding between circRNA-miRNA and miRNA-mRNA. According to Figures 6B–6D and Table S3, circTshz2-mmu-miR-326-3p-Th with the largest fold value and larger binding energy was selected for validation experiment. As expected, mmu-miR-326-3p mimics, but not mimics NC, significantly suppressed luciferase activity of wild-type circTshz2 and Th; this inhibition effect disappeared when the predicted binding sites were mutant (Figures 6E and 6F). Therefore, circRNAs could involve in the process of EX mitigating the progression of PD through the ceRNA mechanism, such as circTshz2-mmu-miR-326-3p-Th.

## 4. Discussion

The neuropathology of PD is the progressive loss of dopaminergic neurons in the substantia nigra, resulting in decreased dopamine levels in the striatum and the accumulation of SNCA resulting in Lewy bodies (Beach et al., 2014; Eschbach and Danzer 2014). These two conditions hallmark the onset of PD, a characteristic combination of motor symptoms (Meder et al., 2019). As early as 2016, the number of PD patients has exceeded 10 million, causing PD to become the second most common neurodegenerative disease in the world (Veys et al., 2019). However, PD cannot be prevented, alleviated, or cured. Worryingly, with the aging of China’s population structure, more and more people will suffer from PD, and the social burden will become more serious. It is extremely urgent and necessary to find effective intervention methods for PD. EX alters neuromodulators, brain chemistry, neurotransmitters, and hypothalamic-pituitary function, providing the brain with a feeling of “energy” and improving cognitive function and quality of life (Garber et al., 2011; Moylan et al., 2013; Klaperski et al., 2014). Therefore, EX can contribute to improved physiology in the case of neurogenic diseases. A study reviewed the effects of EX on fibromyalgia and found that EX may reduce the intensity of pain, improve physical function, and slightly reduce the degree of fatigue (Bidonde et al., 2017). Also, EX has a positive neurobiological effect on patients with schizophrenia (Maurus et al., 2019). Although some studies have shown that substantia nigra-striatal dopamine exhaustion may be due to the hyperactivity of the hypothalamic nucleus and medial globus pallidus, this manuscript focuses on the improvement of PD by EX, which is a process of an overall change of the organism. In the future, we will explore whether EX can improve PD by changing the function of the hypothalamus. In the present study, a PD mouse model was established, and behavioral experiments along with HE staining confirmed that EX could improve the motor function and pathological structure of mice. We found that in the PD group, neurons contracted and were deeply stained while cytoplasmic boundaries were lost, indicating neuron loss, which is typical pathological damage of PD. The phenomenon was also observed in the HE results described by a previous study that the cell size of neurons contracted and neuronal cells were significantly reduced, showing obvious pathological damage of PD (Zhai et al., 2020). Farag et al. (2009) also noted significant neuronal loss in PD (Farag et al., 2009). Importantly, it is worth noting that EX significantly reduced neuronal loss during HE staining. Therefore, histopathology confirmed that EX can improve the pathology of PD.

The circRNA expression profile during the improvement of PD by EX has not been reported, yet the function and molecular mechanisms of EX in PD have been partially examined in our study. We obtained the circRNA expression profile and identified 142 DEcircRNAs between the PD group and the EX-PD group. Compared with the PD group, 89 DEcircRNAs were upregulated and 53 DEcircRNAs were downregulated in the EX-PD group. Unlike our study, in the study of Hanan et al. (2020), there were 6 DEcircRNAs significant increase and 18 DEcircRNAs significant decrease in the healthy human substantia nigra tissue, compared to PD group, the number of upregulation of DEcircRNAs in the healthy group was less than the number that was downregulated. The reason for this difference may be the difference in the design of the two studies. In this study, the DEcircRNA was induced by EX, while Hana et al., (2020) studied the DEcircRNA caused by PD. The other reason for this difference may be that EX triggers the transcription of more genes to participate in the PD recovery process, or it may be due to sampling heterogeneity and differences between species.

In addition, KEGG analysis showed that the DEcircRNAs were mainly involved in ECM-receptor interactions, PD, dopaminergic synapse, and the calcium signaling pathway. ECM is composed of a complex mixture of structural and functional macromolecules, which determines the important role of ECM-receptor interactions in the maintenance of the microenvironmental pathways of the structure and function of cells and tissues (Wang et al., 2020). The ECM-receptor interaction is also indispensable in the protection of neuronal cells and their microenvironment. Similarly, Botta-Orfila et al. (2014) identified differentially expressed miRNAs (DEmiRNAs) in blood associated with PD and found that these DEmiRNAs were also involved in the ECM-receptor interaction pathway, interestingly, miR-532-5p was a member of the DEmiRNAs, which also predicted the target miRNA of circLrpib in our study (Figure 5) (Botta-Orfila et al., 2014). Therefore, EX may regulate the expression of circRNA by sponge miRNA to maintain the stability of ECM-receptor interactions, thereby reducing the impact of PD on the nerves. Moreover, many studies have shown that the calcium signaling pathway involved in intracellular calcium homeostasis and the dopaminergic synapse pathway have critical effects on neuronal function and survival (Cali et al., 2014; Segura-Aguilar et al., 2014; Zaichick et al., 2017; Zhai et al., 2019). No matter which factor is destroyed, it will cause PD pathway obstacles, eventually leading to PD. Thus, we speculated that EX regulated the expression of circRNA to repair the key pathways in the PD, thereby improving physiological status and slowing down disease progression.

In our study, verification of RT-qPCR showed that only circHivep2, circTshz2, and circZfp827 were expressed as expected. The host gene of circHivep2 is human immunodeficiency virus type I enhancer binding protein 2 (HIVEP2) of chromosome 10, which is a dopaminergic transcription factor associated with substance use disorders of dopaminergic neurons in both mice and humans (Zhao et al., 2019). The circHivep2 was predicted to be only one target of miR-3064-5p, which was also associated with neurons. The miR-3064-5p plays an antiangiogenic role, and antiangiogenic inhibitors have been shown to directly regulate MPTP-induced inflammation and dopaminergic neuron loss in a mouse model of PD (Patel et al., 2011; Zhang et al., 2020). Moreover, miR-330-5p and miR-326-3p were predicted as potential targets for circTshz2. Cai et al. (2017b) established that competitive inhibition of miR-330-5p / miR-326-3p expression during the early onset of AD can promote the formation of hippocampal neuronal dendrites. In addition, miR-330-5p and miR-326-3p were predicted to developmentally regulate hippocampal neurons (Cohen et al., 2014). Inhibition of miR-326-3p decreased the number of cells entering the G2/M phase along with the expression of cyclin D1 (Mo et al., 2020). Importantly, Th was predicted as potential target genes for circTshz2, and the distribution of Th in the central and peripheral nervous system often corresponds to that of the neuronal degeneration in idiopathic PD (Haavik et al., 1997). Furthermore, Col24a1 and Th were predicted as potential target genes for circZfp827. Similarly, the targets miR-207 and miR-149-3p of circZfp827 are also involved in neuronal regulation. Exosomal miR-207 alleviated symptoms of depression in stressed mice by inhibiting NF-κB signaling in astrocytes, and the dysregulation of miR-207 in the hippocampus may be related to the cognitive dysfunction caused by intermittent hypoxia (Gao et al., 2017; Li et al., 2020). Therefore, the above results support the involvement of circHivep2, circTshz2, and circZfp827 in the progression of PD by regulating miRNA-targeted mRNA. Combined with the results of RT-qPCR, we speculated that EX increased/inhibited the expression of circHivep2, circTshz2, and circZfp827 and released miRNAs through a ceRNA mechanism to promote the development of PD. However, few studies have been reported on these three DEcircRNAs, their functions are still unclear, and we have not been able to retrieve supporting literature about their direct involvement in PD pathology. The preliminary determination that circHivep2, circTshz2, and circZfp827 are involved in the progress of PD is both the innovation and the regret of this study. In the future, we will further explore these three circRNAs function and significance in the PD process.

In conclusion, our research revealed that EX improved motor dysfunction and pathological features of model mice of PD, and obtained the circRNA expression profile in this process. A total of 142 circRNAs were abnormally expressed after EX intervention, and there were 89 upregulated and 53 downregulated circRNAs in the EX-PD group compared with the PD group. Among them, the expression patterns of circZfp827, circHivep2, and circTshz2 were consistent with RNA-seq, and these three DEcircRNAs may be participated in EX to improve PD progression through the ceRNA mechanism. Overall, this article provides new evidence and molecular targets for EX therapy for PD. In the future, we will test the practical significance and medical value of EX and circZfp827, circHivep2, and circTshz2 in PD patients in clinical samples.

## Additional files

**Table S1:** Primers information used in this study.

Name	Sequences (5′–3′)
GAPDH-F	CAAAATGGTGAAGGTCGGTGT
GAPDH-R	GAGGTCAATGAAGGGGTCGTT
mmu-circHivep2-F	ACATTCTCTCAACAGCCAGCC
mmu-circHivep2-R	GGAGATTGCATTTCCTACCTGGA
mmu-circZfp827-F	AAGCTGAAAGACCCCTCCGA
mmu-circZfp827-R	TGCCTACTAACATCTGACAGCTT
mmu-circTshz2-F	CAAGTCACCCGAACACCACT
mmu-circTshz2-R	CTGGTGTTGAGCAACGGAGC
mmu-circLrp1b-F	TTGTGGAAACTTCTTGTTCTGGAC
mmu-circLrp1b-R	GAGATGCCATTGGGCCATGT
mmu-circSv2b-F	CGCCACGATCAACTTTACCA
mmu-circSv2b-R	GCTGAAGCCCCAGCTTATCG
siRNA-1-circHivep2-F	UAGAUUGGUGAAUUCACCUGATT
siRNA-1-circHivep2-R	UCAGGUGAAUUCACCAAUCUATT
siRNA-2-circHivep2-F	GCCAGCCAGUAGAUUGGUGAATT
siRNA-2-circHivep2-R	UUCACCAAUCUACUGGCUGGCTT
siRNA-1-circTshz2-F	UUCUGCAGGGUAUGCCCAGGATT
siRNA-1-circTshz2-R	UCCUGGGCAUACCCUGCAGAATT
siRNA-2-circTshz2-F	UGAAGAAUAGUUCUGCAGGGUTT
siRNA-2-circTshz2-R	ACCCUGCAGAACUAUUCUUCATT
siRNA-1-circZfp827-F	UUCUCAUGAAGCUGUCAGAUGTT
siRNA-1-circZfp827-R	CAUCUGACAGCUUCAUGAGAATT
siRNA-2-circZfp827-F	GCUGUCAGAUGUUAGUAGGCATT
siRNA-2-circZfp827-R	UGCCUACUAACAUCUGACAGCTT
mmu-Col24a1-F	TGAATTTTACCCTGATGCCACG
mmu-Col24a1-R	CATGTCAAGCACTGCGTTGT
mmu-Tph1-F	GCCTGTTACACATCGAGTCCC
mmu-Tph1-R	ACAGTCTCCATAACGTCTTCCTT
mmu-Th-F	GTCTCAGAGCAGGATACCAAGC
mmu-Th-R	CTCTCCTCGAATACCACAGCC

**Table S2:** Clean data quality control and statistics.

Samples	Total reads	Reads filter (%)	Total bases	Bases filter (%)	GC (%)	Mapped rate (%)
M1	98777574	0.947	14892252983	0.946	45.5	0.915
M2	116864136	0.952	17619381925	0.951	45	0.925
M3	87604768	0.947	13207460120	0.945	46	0.923
Z1	94325600	0.952	14221402578	0.951	45	0.923
Z2	55639460	0.956	8388419770	0.954	45	0.937
Z3	68269528	0.960	10293099636	0.958	44	0.946

M means sample collected from PD mice without aerobic exercise. Z means sample collected from PD mice with aerobic exercise.

**Table S3:** The predicted target genes for five DEcircRNAs using MiRanda and TargetScan.

miRNA ID	mRNA ID	miRNA mRNA RNAhybrid	Style	miRNA ID	circRNA ID	miRNA circRNA RNAhybrid	Style
mmu-miR-7222-3p	Nxph4	0.0210	down	mmu-miR-7222-3p	chr8_79076538_79060250_+16288-Zfp827	0.1694	down
mmu-miR-3064-5p	Bub1b	0.0169	down	mmu-miR-3064-5p	chr8_79076538_79060250_+16288-Zfp827	0.3903	down
mmu-miR-149-3p	Bglap3	0.0004	down	mmu-miR-149-3p	chr8_79076538_79060250_+16288-Zfp827	0.0231	down
mmu-miR-3091-3p	Kcnj14	0.0363	down	mmu-miR-3091-3p	chr8_79076538_79060250_+16288-Zfp827	0.2705	down
mmu-miR-7030-5p	Nxph4	0.0091	down	mmu-miR-7030-5p	chr8_79076538_79060250_+16288-Zfp827	0.2584	down
mmu-miR-7030-5p	Prph	0.0075	down	mmu-miR-7030-5p	chr8_79076538_79060250_+16288-Zfp827	0.2584	down
mmu-miR-7659-3p	Slc6a3	0.2746	down	mmu-miR-7659-3p	chr8_79076538_79060250_+16288-Zfp827	0.0532	down
mmu-miR-1943-5p	Bglap3	0.0013	down	mmu-miR-1943-5p	chr8_79076538_79060250_+16288-Zfp827	0.2870	down
mmu-miR-6943-5p	Nr4a2	0.1478	down	mmu-miR-6943-5p	chr8_79076538_79060250_+16288-Zfp827	0.2061	down
mmu-miR-6943-5p	Stab2	0.0070	down	mmu-miR-6943-5p	chr8_79076538_79060250_+16288-Zfp827	0.2061	down
mmu-miR-3058-5p	Col24a1	0.0778	down	mmu-miR-3058-5p	chr8_79076538_79060250_+16288-Zfp827	0.1229	down
mmu-miR-207	Popdc3	0.0535	down	mmu-miR-207	chr8_79076538_79060250_+16288-Zfp827	0.1884	down
mmu-miR-5621-5p	Kcnj14	0.0305	down	mmu-miR-5621-5p	chr8_79076538_79060250_+16288-Zfp827	0.0825	down
mmu-miR-7024-5p	Isl1	0.0716	down	mmu-miR-7024-5p	chr8_79076538_79060250_+16288-Zfp827	0.0201	down
mmu-miR-7024-5p	Stc2	0.0164	down	mmu-miR-7024-5p	chr8_79076538_79060250_+16288-Zfp827	0.0201	down
mmu-miR-7083-3p	Stc2	0.0442	down	mmu-miR-7083-3p	chr8_79076538_79060250_+16288-Zfp827	0.3159	down
mmu-miR-470-5p	Isl1	0.4927	down	mmu-miR-470-5p	chr8_79076538_79060250_+16288-Zfp827	0.1930	down
mmu-miR-5110	Stab2	0.0004	down	mmu-miR-5110	chr8_79076538_79060250_+16288-Zfp827	0.0029	down
mmu-miR-6911-5p	Col24a1	0.0667	down	mmu-miR-6911-5p	chr8_79076538_79060250_+16288-Zfp827	0.0936	down
mmu-miR-7048-5p	Th	0.0199	down	mmu-miR-7048-5p	chr8_79076538_79060250_+16288-Zfp827	0.0823	down
mmu-miR-7048-5p	Prph	0.0326	down	mmu-miR-7048-5p	chr8_79076538_79060250_+16288-Zfp827	0.0823	down
mmu-miR-3154	Th	0.0125	down	mmu-miR-3154	chr8_79076538_79060250_+16288-Zfp827	0.3253	down
mmu-miR-3154	Nr4a2	0.1333	down	mmu-miR-3154	chr8_79076538_79060250_+16288-Zfp827	0.3253	down
mmu-miR-7081-5p	Th	0.0090	down	mmu-miR-7081-5p	chr8_79076538_79060250_+16288-Zfp827	0.2386	down
mmu-miR-7081-5p	Nefm	0.1670	down	mmu-miR-7081-5p	chr8_79076538_79060250_+16288-Zfp827	0.2386	down
mmu-miR-7038-3p	Col24a1	0.4130	down	mmu-miR-7038-3p	chr8_79076538_79060250_+16288-Zfp827	0.3168	down
mmu-miR-12201-3p	Cysltr2	0.0123	down	mmu-miR-12201-3p	chr8_79076538_79060250_+16288-Zfp827	0.2584	down
mmu-miR-6971-5p	Nxph4	0.0053	down	mmu-miR-6971-5p	chr8_79076538_79060250_+16288-Zfp827	0.0641	down
mmu-miR-6971-5p	Stc2	0.0164	down	mmu-miR-6971-5p	chr8_79076538_79060250_+16288-Zfp827	0.0641	down
mmu-miR-3075-3p	8430408G22Rik	0.0393	down	mmu-miR-3075-3p	chr8_79076538_79060250_+16288-Zfp827	0.0522	down
mmu-miR-6987-5p	Slc6a3	0.1203	down	mmu-miR-6987-5p	chr8_79076538_79060250_+16288-Zfp827	0.0741	down
mmu-miR-6987-5p	Kcnj14	0.1690	down	mmu-miR-6987-5p	chr8_79076538_79060250_+16288-Zfp827	0.0741	down
mmu-miR-7075-5p	Nxph4	0.0090	down	mmu-miR-7075-5p	chr8_79076538_79060250_+16288-Zfp827	0.1061	down
mmu-miR-7018-3p	Nefm	0.0488	down	mmu-miR-7018-3p	chr8_79076538_79060250_+16288-Zfp827	0.0481	down
mmu-miR-6985-5p	Nefm	0.0367	down	mmu-miR-6985-5p	chr8_79076538_79060250_+16288-Zfp827	0.0888	down
mmu-miR-7053-5p	Nxph4	0.0120	down	mmu-miR-7053-5p	chr8_79076538_79060250_+16288-Zfp827	0.3796	down
mmu-miR-7077-5p	Kcnj14	0.0312	down	mmu-miR-7077-5p	chr8_79076538_79060250_+16288-Zfp827	0.0515	down
mmu-miR-8100	Th	0.0018	down	mmu-miR-8100	chr8_79076538_79060250_+16288-Zfp827	0.1296	down
mmu-miR-6919-5p	Kcnj14	0.1186	down	mmu-miR-6919-5p	chr8_79076538_79060250_+16288-Zfp827	0.0599	down
mmu-miR-7226-5p	Lrrc52	0.0263	down	mmu-miR-7226-5p	chr8_79076538_79060250_+16288-Zfp827	0.1041	down
mmu-miR-7023-5p	Slc6a3	0.1742	down	mmu-miR-7023-5p	chr8_79076538_79060250_+16288-Zfp827	0.0387	down
mmu-miR-7023-5p	Col24a1	0.0712	down	mmu-miR-7023-5p	chr8_79076538_79060250_+16288-Zfp827	0.0387	down
mmu-miR-7023-5p	Isl1	0.1968	down	mmu-miR-7023-5p	chr8_79076538_79060250_+16288-Zfp827	0.0387	down
mmu-miR-6924-5p	Isl1	0.1587	down	mmu-miR-6924-5p	chr8_79076538_79060250_+16288-Zfp827	0.3307	down
mmu-miR-6924-5p	8430408G22Rik	0.0871	down	mmu-miR-6924-5p	chr8_79076538_79060250_+16288-Zfp827	0.3307	down
mmu-miR-6934-3p	Adm	0.0919	down	mmu-miR-6934-3p	chr8_79076538_79060250_+16288-Zfp827	0.0533	down
mmu-miR-6934-3p	Col24a1	0.2511	down	mmu-miR-6934-3p	chr8_79076538_79060250_+16288-Zfp827	0.0533	down
mmu-miR-3064-5p	Sh3rf2	0.208098	up	mmu-miR-3064-5p	chr10_14066684_14004201_+62483-Hivep2	0.023378	up
mmu-miR-3064-5p	Tph1	0.415318	up	mmu-miR-3064-5p	chr10_14066684_14004201_+62483-Hivep2	0.023378	up
mmu-miR-3064-5p	Hs3st2	0.383591	up	mmu-miR-3064-5p	chr10_14066684_14004201_+62483-Hivep2	0.023378	up
mmu-miR-3064-5p	Lrrc32	0.050883	up	mmu-miR-3064-5p	chr10_14066684_14004201_+62483-Hivep2	0.023378	up
mmu-miR-3064-5p	Nags	0.031333	up	mmu-miR-3064-5p	chr10_14066684_14004201_+62483-Hivep2	0.023378	up
mmu-miR-3064-5p	Smoc2	0.296494	up	mmu-miR-3064-5p	chr10_14066684_14004201_+62483-Hivep2	0.023378	up
mmu-miR-330-5p	Th	0.027769	down	mmu-miR-330-5p	chr2_169886586_169883526_+3060-Tshz2	0.143068	down
mmu-miR-6915-5p	Prph	0.026982	down	mmu-miR-6915-5p	chr2_169886586_169883526_+3060-Tshz2	0.087382	down
mmu-miR-7072-5p	Nxph4	0.011177	down	mmu-miR-7072-5p	chr2_169886586_169883526_+3060-Tshz2	0.151737	down
mmu-miR-7117-5p	Popdc3	0.271656	down	mmu-miR-7117-5p	chr2_169886586_169883526_+3060-Tshz2	0.09658	down
mmu-miR-3547-5p	Nxph4	0.000227	down	mmu-miR-3547-5p	chr2_169886586_169883526_+3060-Tshz2	0.019554	down
mmu-miR-3547-5p	Tmc5	0.002207	down	mmu-miR-3547-5p	chr2_169886586_169883526_+3060-Tshz2	0.019554	down
mmu-miR-3547-5p	Kcnj14	0.02742	down	mmu-miR-3547-5p	chr2_169886586_169883526_+3060-Tshz2	0.019554	down
mmu-miR-6907-5p	Tmc5	0.072564	down	mmu-miR-6907-5p	chr2_169886586_169883526_+3060-Tshz2	0.381032	down
mmu-miR-7074-5p	Tmc5	0.00233	down	mmu-miR-7074-5p	chr2_169886586_169883526_+3060-Tshz2	0.100129	down
mmu-miR-7074-5p	Prph	0.001958	down	mmu-miR-7074-5p	chr2_169886586_169883526_+3060-Tshz2	0.100129	down
mmu-miR-1962	Bub1b	0.006412	down	mmu-miR-1962	chr2_169886586_169883526_+3060-Tshz2	0.133816	down
mmu-miR-1962	Kcnj14	0.219149	down	mmu-miR-1962	chr2_169886586_169883526_+3060-Tshz2	0.133816	down
mmu-miR-7030-5p	Nxph4	0.009068	down	mmu-miR-7030-5p	chr2_169886586_169883526_+3060-Tshz2	0.020618	down
mmu-miR-7030-5p	Prph	0.007489	down	mmu-miR-7030-5p	chr2_169886586_169883526_+3060-Tshz2	0.020618	down
mmu-miR-1955-5p	Popdc3	0.220511	down	mmu-miR-1955-5p	chr2_169886586_169883526_+3060-Tshz2	0.407988	down
mmu-miR-5133	Th	0.054818	down	mmu-miR-5133	chr2_169886586_169883526_+3060-Tshz2	0.156608	down
mmu-miR-5133	Stab2	0.036899	down	mmu-miR-5133	chr2_169886586_169883526_+3060-Tshz2	0.156608	down
mmu-miR-6955-5p	Kcnj14	0.07244	down	mmu-miR-6955-5p	chr2_169886586_169883526_+3060-Tshz2	0.263939	down
mmu-miR-6931-5p	Nxph4	0.008368	down	mmu-miR-6931-5p	chr2_169886586_169883526_+3060-Tshz2	0.016892	down
mmu-miR-326-3p	Th	0.07732	down	mmu-miR-326-3p	chr2_169886586_169883526_+3060-Tshz2	0.050376	down
mmu-miR-6943-5p	Nr4a2	0.147846	down	mmu-miR-6943-5p	chr2_169886586_169883526_+3060-Tshz2	0.208159	down
mmu-miR-6943-5p	Stab2	0.007027	down	mmu-miR-6943-5p	chr2_169886586_169883526_+3060-Tshz2	0.208159	down
mmu-miR-470-5p	Isl1	0.492706	down	mmu-miR-470-5p	chr2_169886586_169883526_+3060-Tshz2	0.294493	down
mmu-miR-5110	Stab2	0.000362	down	mmu-miR-5110	chr2_169886586_169883526_+3060-Tshz2	0.031809	down
mmu-miR-7081-5p	Th	0.009045	down	mmu-miR-7081-5p	chr2_169886586_169883526_+3060-Tshz2	0.109657	down
mmu-miR-7081-5p	Nefm	0.16704	down	mmu-miR-7081-5p	chr2_169886586_169883526_+3060-Tshz2	0.109657	down
mmu-miR-204-3p	Th	0.024804	down	mmu-miR-204-3p	chr2_169886586_169883526_+3060-Tshz2	0.240623	down
mmu-miR-3473e	Kcnj14	0.396675	down	mmu-miR-3473e	chr2_169886586_169883526_+3060-Tshz2	0.395727	down
mmu-miR-3473b	Kcnj14	0.471907	down	mmu-miR-3473b	chr2_169886586_169883526_+3060-Tshz2	0.419929	down
mmu-miR-6987-5p	Slc6a3	0.120275	down	mmu-miR-6987-5p	chr2_169886586_169883526_+3060-Tshz2	0.186479	down
mmu-miR-6987-5p	Kcnj14	0.168954	down	mmu-miR-6987-5p	chr2_169886586_169883526_+3060-Tshz2	0.186479	down
mmu-miR-540-3p	Prph	0.081548	down	mmu-miR-540-3p	chr2_169886586_169883526_+3060-Tshz2	0.480666	down
mmu-miR-709	Slc6a3	0.03024	down	mmu-miR-709	chr2_169886586_169883526_+3060-Tshz2	0.301361	down
mmu-miR-709	Stab2	0.014737	down	mmu-miR-709	chr2_169886586_169883526_+3060-Tshz2	0.301361	down
mmu-miR-106b-3p	Tmc5	0.024053	down	mmu-miR-106b-3p	chr2_169886586_169883526_+3060-Tshz2	0.11347	down
mmu-miR-6368	Tmc5	0.074926	down	mmu-miR-6368	chr2_169886586_169883526_+3060-Tshz2	0.203209	down
mmu-miR-1938	Nxph4	0.089908	down	mmu-miR-1938	chr2_169886586_169883526_+3060-Tshz2	0.111913	down
mmu-miR-12185-5p	Isl1	0.382555	down	mmu-miR-12185-5p	chr2_169886586_169883526_+3060-Tshz2	0.023653	down
mmu-miR-12185-5p	8430408G22Rik	0.162393	down	mmu-miR-12185-5p	chr2_169886586_169883526_+3060-Tshz2	0.023653	down
mmu-miR-7222-5p	Exph5	0.062892	up	mmu-miR-7222-5p	chr2_41113094_41110754_-2340-Lrp1b	0.179368	up
mmu-miR-7222-5p	Tph1	0.361544	up	mmu-miR-7222-5p	chr2_41113094_41110754_-2340-Lrp1b	0.179368	up
mmu-miR-7222-5p	Slc6a20b	0.492442	up	mmu-miR-7222-5p	chr2_41113094_41110754_-2340-Lrp1b	0.179368	up
mmu-miR-696	Mfi2	0.036929	up	mmu-miR-696	chr2_41113094_41110754_-2340-Lrp1b	0.11788	up
mmu-miR-9769-5p	Lyz1	0.115553	up	mmu-miR-9769-5p	chr2_41113094_41110754_-2340-Lrp1b	0.108304	up
mmu-miR-6975-5p	Plekha2	0.074879	up	mmu-miR-6975-5p	chr2_41113094_41110754_-2340-Lrp1b	0.25605	up
mmu-miR-6975-5p	Tph1	0.082795	up	mmu-miR-6975-5p	chr2_41113094_41110754_-2340-Lrp1b	0.25605	up
mmu-miR-6975-5p	Hs3st2	0.091241	up	mmu-miR-6975-5p	chr2_41113094_41110754_-2340-Lrp1b	0.25605	up
mmu-miR-6975-5p	Lrrc32	0.022025	up	mmu-miR-6975-5p	chr2_41113094_41110754_-2340-Lrp1b	0.25605	up
mmu-miR-6975-5p	Slc16a12	0.322382	up	mmu-miR-6975-5p	chr2_41113094_41110754_-2340-Lrp1b	0.25605	up
mmu-miR-6386	Wnt6	0.035637	up	mmu-miR-6386	chr2_41113094_41110754_-2340-Lrp1b	0.119986	up
mmu-miR-532-5p	Wnt6	0.379639	up	mmu-miR-532-5p	chr2_41113094_41110754_-2340-Lrp1b	0.353535	up
mmu-miR-670-3p	Gm5082	0.042866	up	mmu-miR-670-3p	chr2_41113094_41110754_-2340-Lrp1b	0.414485	up
mmu-miR-6959-5p	Cldn2	0.046449	up	mmu-miR-6959-5p	chr7_75149088_75136298_-12790-Sv2b	0.151263	up
mmu-miR-7052-5p	Mfi2	0.0075	up	mmu-miR-7052-5p	chr7_75149088_75136298_-12790-Sv2b	0.012649	up
mmu-miR-7052-5p	Krt80	0.110836	up	mmu-miR-7052-5p	chr7_75149088_75136298_-12790-Sv2b	0.012649	up
mmu-miR-7052-5p	Col6a1	0.00314	up	mmu-miR-7052-5p	chr7_75149088_75136298_-12790-Sv2b	0.012649	up
mmu-miR-3073b-3p	Mfi2	0.456996	up	mmu-miR-3073b-3p	chr7_75149088_75136298_-12790-Sv2b	0.083402	up
mmu-miR-6908-3p	Myom2	0.108262	up	mmu-miR-6908-3p	chr7_75149088_75136298_-12790-Sv2b	0.27356	up
mmu-miR-6908-3p	Mfi2	0.239812	up	mmu-miR-6908-3p	chr7_75149088_75136298_-12790-Sv2b	0.27356	up
mmu-miR-5107-5p	Krt80	0.011591	up	mmu-miR-5107-5p	chr7_75149088_75136298_-12790-Sv2b	0.060083	up
mmu-miR-615-5p	Slc16a12	0.202914	up	mmu-miR-615-5p	chr7_75149088_75136298_-12790-Sv2b	0.005892	up
mmu-miR-6995-5p	Il18r1	0.025983	up	mmu-miR-6995-5p	chr7_75149088_75136298_-12790-Sv2b	0.018591	up
mmu-miR-6995-5p	Svep1	0.02953	up	mmu-miR-6995-5p	chr7_75149088_75136298_-12790-Sv2b	0.018591	up
mmu-miR-6995-5p	Cldn2	0.051102	up	mmu-miR-6995-5p	chr7_75149088_75136298_-12790-Sv2b	0.018591	up
mmu-miR-873a-5p	Tagln	0.025327	up	mmu-miR-873a-5p	chr7_75149088_75136298_-12790-Sv2b	0.101121	up
mmu-miR-873a-5p	Wnt6	0.222104	up	mmu-miR-873a-5p	chr7_75149088_75136298_-12790-Sv2b	0.101121	up
